# The Value of the Biomarkers Neuron-Specific Enolase and S100 Calcium-Binding Protein for Prediction of Mortality in Children Resuscitated After Cardiac Arrest

**DOI:** 10.1007/s00246-022-02899-9

**Published:** 2022-04-16

**Authors:** Johanne Bangshøj, Benedikte Liebetrau, Sebastian Wiberg, Jakob Gjedsted, Jesper Kjærgaard, Christian Hassager, Michael Wanscher

**Affiliations:** 1 Department of Anesthesia and Intensive Care, Bispebjerg Hospital, Copenhagen, Denmark; 2 Department of Cardiology, Copenhagen University Hospital Rigshospitalet, Copenhagen, Denmark; 3 Department of Cardiothoracic Anesthesiology, Copenhagen University Hospital Rigshospitalet, Copenhagen, Denmark

**Keywords:** Children, Cardiac arrest, Prognostication, Neuron-specific enolase, S100b

## Abstract

The aim of the present study was to assess the ability of the biomarkers neuron-specific enolase (NSE) and S100 calcium-binding protein b (S100b) to predict 30 day mortality in children resuscitated from cardiac arrest (CA). It was a prospective observational study at a single tertiary heart centre. Consecutive children were admitted after resuscitated in-hospital and out-of-hospital CA. Levels of NSE and S100b were analyzed from 12 to 24 hours, from 24 to 48 hours, and from 48 to 72 hours after admission. The primary endpoint was 30-day mortality. Differences in biomarker levels between survivors and non-survivors were analyzed with the Mann-Whitney U test. Receiver operating characteristics (ROC) curves were applied to assess the predictive ability of the biomarkers and the areas under the ROC curves (AUC) were presented. A total of 32 resuscitated CA patients were included, and 12 (38%) patients died within 30 days after resuscitation. We observed significantly higher levels of NSE and S100b in non-survivors compared to survivors at all timepoints from 12 to 72 hours after CA. NSE achieved AUCs from 0.91–0.98 for prediction of 30 day mortality, whereas S100b achieved AUCs from 0.93–0.94. An NSE cut-off of 61 μg/L sampled between 12–24 hours from admission achieved a sensitivity of 80% and a specificity of 100% for prediction of 30 day mortality. In children resuscitated from CA, the biomarkers NSE and S100b appear to be solid predictors of mortality after 30 days.

## Introduction

Survival rates in children with cardiac arrest (CA) are poor, even if return of spontaneous circulation (ROSC) is achieved [1, 2]. Survival rates are reported as low as 30% after in-hospital CA (IHCA) and 8% after out-of-hospital CA (OHCA) [1, 3]. The primary cause of severe morbidity and mortality after resuscitated CA is neurological injury occurring as part of the post-CA syndrome [1, 4].

In patients remaining unconscious after ROSC, the decision whether to continue treatment or not, and for how long, is notoriously difficult [5, 6], as prognosis depends on a multitude of factors, including the cause of CA, time to ROSC, individual patient characteristics such as age and comorbidities. No present test or imaging are sufficiently accurate to be recommended as a stand-alone prognostication tool by contemporary guidelines that advocate for a multimodal prognostication strategy [7]. However, precision of individual prognostication tools after resuscitated CA has been sparsely investigated in children. Furthermore, a number of currently applied prognostic tests such as neurologic examination and electroencephalography (EEG) may be affected by sedatives which are frequently used in the post-resuscitation phase [8, 9]. As such, a biomarker with a high precision for prediction of poor outcome after resuscitated CA in children could be of great benefit.

Neuron-specific enolase (NSE) and S100 calcium-binding protein b (S100b) are relatively novel markers of cerebral injury [10, 11]. In adults, several studies have shown NSE to be a predictor of death and poor neurological outcome after CA [6, 12, 13]. A similar association has been reported for S100b, albeit less strong compared to NSE [5, 14]. Whether NSE and/or S100b are useful for prediction of outcome after CA in children has been sparsely investigated [1, 3, 15]. Accordingly, the aim of the present paper was to investigate the predictive ability of NSE and S100b in children resuscitated from CA for mortality and neurological outcome.

## Materials and Methods

### Study Design and Setting

The present study is a prospective analysis from a tertiary heart center. Consecutive patients younger than 18 years were included in the time period from February 2011 to December 2021. Patients being resuscitated from IHCA as well as patients being admitted after resuscitated OHCA were included. CA was defined as external chest compressions for one minute or more [1, 3]. Only patients remaining unconscious after ROSC were included. Patient characteristics were collected from electronic patient charts. All endpoints and analyses were defined a priori. The study was approved by the center’s institutional review board and determined to meet the requirements of health surveillance and quality control. Biomarkers were measured as part of clinical routine, and due to the observational nature of the study, a waiver of informed consent was granted.

### Patients

After resuscitation, all patients were admitted to an intensive care unit (ICU) for post-resuscitation care. On admission to the ICU, an arterial blood gas was drawn and analyzed. As part of clinical routine, blood was analyzed for NSE and S100b daily from 12 to 72 h after admission. Serial NSE and S100b analyses were being adopted gradually as routine procedure at our institution during the inclusion period. Accordingly, blood for analysis of NSE and S100b was frequently drawn at fixed rounds, simultaneous with blood for analyses of other biochemistry. Thus, NSE and S100b levels were not measured at specific timepoints after ROSC, but rather in the following time periods: 12 to 24 h, 24 to 48 h, and 48 to 72 h after ROSC (or ECMO initiation), Post-resuscitation care was provided according to institutional standard procedures following contemporary guidelines. Further diagnostic examinations, including EEG, somatosensory evoked potentials (SSEP) or cerebral imaging, were not routinely used but were initiated at attending physicians’ discretion.

### Biomarker Analysis

Analyses of NSE were performed with the Cobas 8000, e602 module, using an electrochemiluminescence immunoassay (ECLIA) kit (Roche Diagnostics), with a range from 0.1 to 370 μg/L and a between-run precision at 11 μg/L and 85 μg/L of 8% and 7%, respectively. Analyses of S100b were performed with the Cobas 8000, e602 module, using an ECLIA kit (Roche Diagnostics), with a range from 0.02 to 39 μg/L and a between-run precision at 0.09 μg/L and 3.3 μg/L of 6%.

The department of Biochemistry applied specific equations to correct measured levels of NSE for hemolysis.

### Outcome Measurements

The primary outcome was 30-day mortality. The secondary outcome was neurologic outcome after 180 days, assessed by the pediatric cerebral performance category (PCPC) score [16]. Neurological outcome was dichotomized into good neurological outcome (a PCPC score of one to three) or poor neurological outcome (a PCPC score of four to six) equivalent to severe disability, coma or death [3, 15]. Outcomes were adjudicated by review of medical files by two of the authors of this manuscript.

### Statistical Analyses

Assuming a mean (SD) NSE level of 90 (60) µg/L after 48 h in non-survivors and a mean (SD) NSE level of 25 (60) µg/L after 48 h in survivors, the study would achieve a power of 0.84 at a significance level of 0.05 if 30 patients were included (sample size calculation completed with the non-parametric Mann–Whitney U-test). The assumed NSE levels were based on an adult population with out-of-hospital CA [17]. Categorical variables were presented as numbers (*n*) and percentages (%) and differences between strata were compared with the Chi-Square test or Fisher’s Exact test as appropriate. Continuous variables were presented as mean ± standard deviation (SD) if normally distributed or as median (inter-quartile range, IQR) if skewed and differences between strata were compared with the Student’s *t*-test or the Mann–Whitney U test as appropriate. Baseline characteristics were presented stratified by 30-day survival. Median levels of NSE and S100b were presented at each time point after stratification by 30-day survival. To assess the predictive ability of NSE and S100b, receiver operating characteristics (ROC) were applied, and the area under the curves (AUC) were presented. The Hosmer and Lemeshow test were applied to assess the goodness-of-fit for the models. In addition, for a set specificity of 100% (i.e., a false-positive rate of 0), we presented the cut-off values with corresponding predictive values for each biomarker measurement. All tests were two-sided with a significance level of 0.05. Statistical analyses are done with SAS Enterprise Guide software, version 7.1.

## Results

A total of 32 patients were included in the study. A total of 10 (31%) patients were female, median age was 12 (4–16) years with a range from 0 to 17 years. Within the first 30 days from admission 12 (38%) patients died. Non-survivors had a significantly higher level of blood glucose after admission compared to survivors (Table 1). Non-survivors had significantly higher lactate levels 12 h after admission compared to survivors. We found no other significant differences in baseline characteristics between non-survivors and survivors (Table 1).

**Table 1:** Baseline characteristics, stratified by 30-day mortality

	Survivors 20 (63%)	Non-survivors 12 (38%)	*p*
Age, median (IQR)	15 (6–16)	7 (2–15)	0.19
Female sex, *n* (%)	6 (30%)	4 (33%)	0.84
Out-of-hospital cardiac arrest, *n* (%)	16 (80%)	8 (67%)	0.43
Location, *n* (%)			
Home	5 (25%)	3 (25%)	
Public	11 (55%)	5 (42%)	
Hospital	4 (20%)	4 (33%)	0.67
Cause, *n* (%)			
Asphyxia	9 (45%)	5 (42%)	
Cardiac	3 (15%)	2 (17%)	
Other	8 (40%)	5 (42%)	0.98
Primary shockable rhythm, *n* (%)	9 (45%)	2 (25%)	0.45
Witnessed event, *n* (%)	18 (90%)	7 (58%)	0.07
Bystander CPR, *n* (%)	5 (25%)	6 (50%)	0.25
Minutes to ROSC or ECMO, median (IQR)	21 (10–97)	45 (20–60)	0.19
Extracorporeal membrane oxygenation, *n* (%)	9 (45%)	6 (50%)	> 0.99
Biomarkers, median (IQR)			
First pH	6.9 (6.6–7.3)	6.8 (6.6–6.9)	0.10
pH after 12 h	7.4 (7.3–7.5)	7.4 (7.3–7.5)	0.92
First lactate (mmol/L)	12 (7.1–18)	15 (14–20)	0.08
Lactate after 12 h (mmol/L)	1.4 (1.1.–3.3)	3.6 (2.7–10)	0.04
First blood glucose (mmol/L)	9.7 (7.1–16)	20 (14–23)	0.006

*IQR* interquartile range, *CPR* cardiopulmonary resuscitation, *ROSC* return of spontaneous circulation, *ECMO* extracorporeal membrane oxygenation

No patients died between 30 and 180 days from admission. After 180 days, 14 (44%) patients had a PCPC score of four or higher, i.e., a poor neurologic outcome including death.

### Neuron-Specific Enolase Levels

**Fig. 1:**
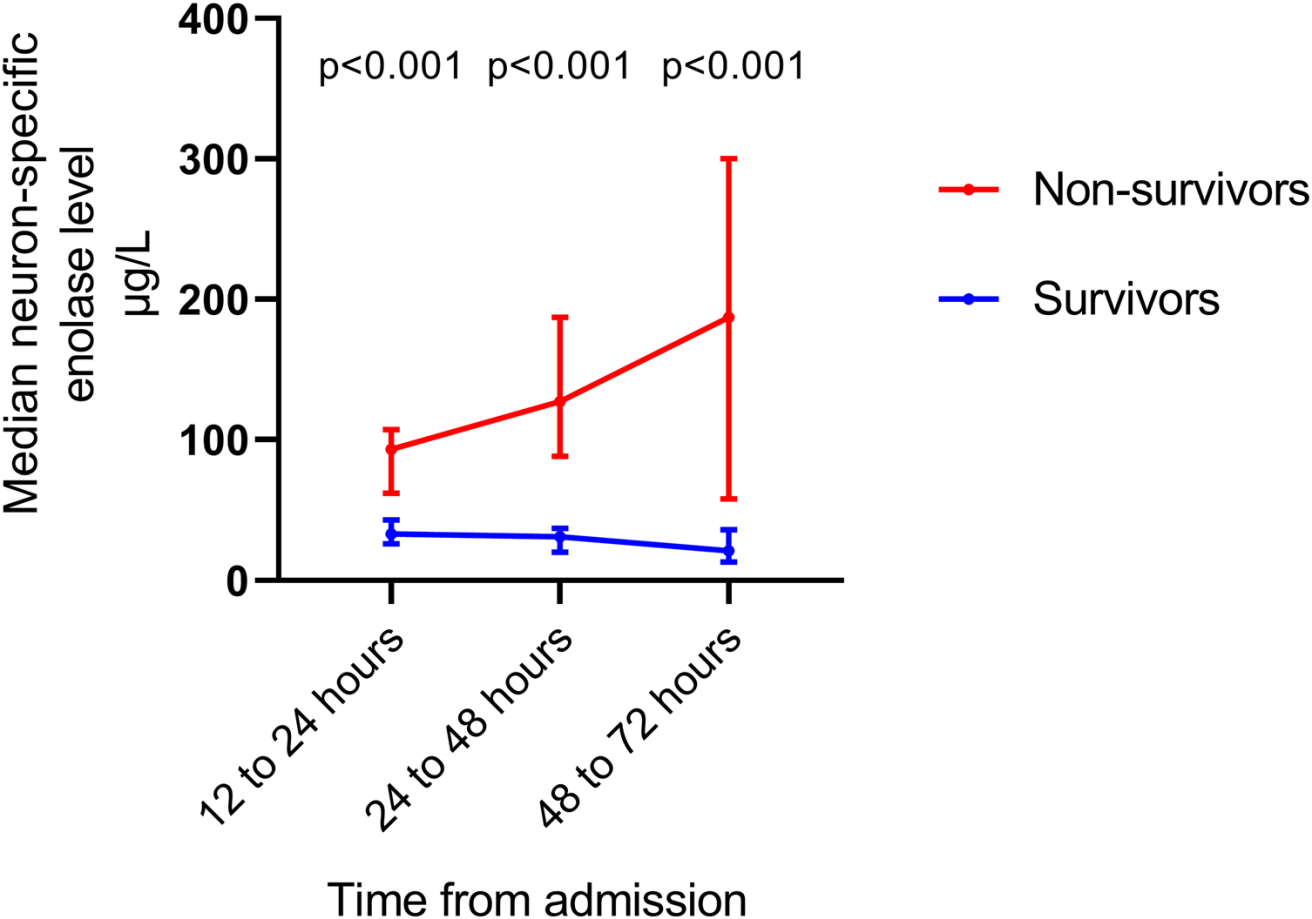
Median (interquartile range) levels of neuron-specific enolase stratified by 30-day mortality

A total of 29 (91%) had NSE measured between 12 and 24 h, 29 (91%) had NSE measured between 24 and 48 h, and 21 (66%) had NSE measured between 48 and 72 h. While decreasing in survivors, median NSE levels increased from 12 to 72 h from admission in non-survivors (*p* < 0.001). Median NSE levels were significantly higher in non-survivors versus survivors at all time points (Fig. 1).

The AUC for prediction of 30-day mortality was 0.91 (0.75–1.0) for NSE measured after 12 to 24 h, 0.97 (0.92–1.0) for NSE measured after 24 to 48 h, and 0.98 (0.93–1.0) for NSE measured after 48 to 72 h. The Hosmer and Lemeshow goodness-of-fit tests were insignificant for all three models (*p* = 0.27–0.99).

For a set specificity of 100% (i.e., a false-positive rate of 0), the cut-off levels of NSE were 61 μg/L when measured between 12 and 24 h, 98 μg/L when measured between 24 and 48 h, and 59 μg/L when measured between 48 and 72 h (Table 2).

**Table 2:** Cut-off values and predictive values of biomarkers for prediction of 30-day mortality at a set specificity of 100% (i.e., a false-positive rate of 0%)

	*n*	Cut-off (μg/L)	Specificity (%)	Sensitivity (%)	PPV (%)	NPV (%)
NSE between 12 and 24 h	29	61	100	80	100	90
NSE between 24 and 48 h	29	98	100	50	100	79
NSE between 48 and 72 h	21	59	100	67	100	88
S100b between 12 and 24 h	31	2.0	100	73	100	87
S100b between 24 and 48 h	31	3.3	100	27	100	71
S100b between 48 and 72 h	22	0.98	100	57	100	83

*PPV* positive predictive value, *NPV* negative predictive value, *NSE* neuron-specific enolase

The AUC for prediction of poor neurologic outcome after 180 days was 0.90 (0.76–1.0) for NSE measured after 12 to 24 h, 0.92 (0.80–1.0) for NSE measured after 24 to 48 h, and 0.88 (0.72–1.0) for NSE measured after 48 to 72 h. The cut-offs with corresponding predictive values are presented in Table 3.

**Table 3:** Cut-off values and predictive values of biomarkers for prediction of poor neurologic outcome after 180 days (defined as a Pediatric Cerebral Performance Category score of four to six) at a set specificity of 100% (i.e., a false-positive rate of 0%)

	*n*	Cut-off (μg/L)	Specificity (%)	Sensitivity (%)	PPV (%)	NPV (%)
NSE between 12 and 24 h	29	56	100	75	100	85
NSE between 24 and 48 h	29	98	100	42	100	71
NSE between 48 and 72 h	21	59	100	50	100	76
S100b between 12 and 24 h	31	2.0	100	62	100	78
S100b between 24 and 48 h	31	3.3	100	23	100	64
S100b between 48 and 72 h	22	0.98	100	44	100	72

*PPV* positive predictive value, *NPV* negative predictive value, *NSE* neuron-specific enolase

### S100b Levels

**Fig. 2:**
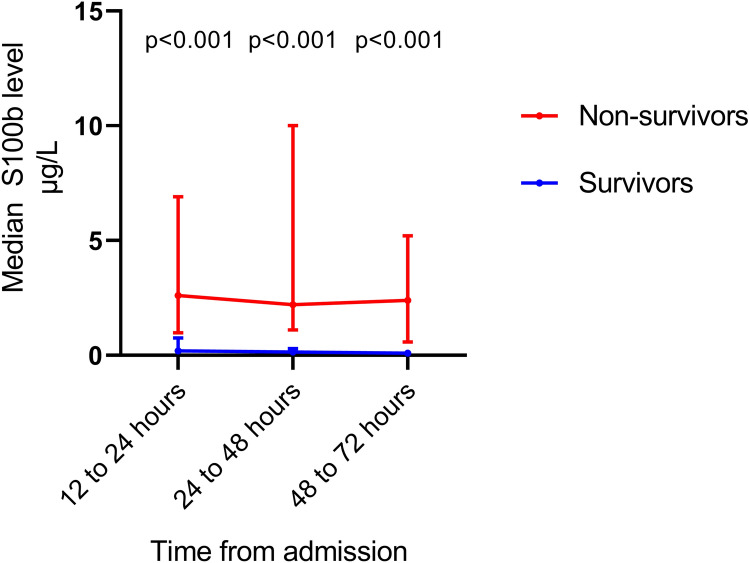
Median (interquartile range) levels of S100b stratified by 30-day mortality

A total of 31 (97%) had S100b measured between 12 and 24 h, 31 (97%) had S100b measured between 24 and 48 h, and 22 (69%) had S100b measured between 48 and 72 h. Median levels of S100b at all time points were significantly higher in non-survivors versus survivors (Fig. 2). The area under the ROC curves for prediction of 30-day mortality was 0.93 (0.84–1.0) for S100b measured after 12 to 24 h, 0.94 (0.86–1.0) for S100b measured after 24 to 48 h, and 0.94 (0.85–1.0) for S100b measured after 48 to 72 h. The Hosmer and Lemeshow goodness-of-fit tests were insignificant for all three models (*p* = 0.25–0.84).

For a set specificity of 100% (i.e., a false-positive rate of 0), the cut-off levels of S100b were 2.0 μg/L when measured between 12 and 24 h, 3.3 μg/L when measured between 24 and 48 h, and 0.98 μg/L when measured between 48 and 72 h (Table 2).

The AUC for prediction of poor neurologic outcome after 180 days was 0.89 (0.77–1.0) for S100b measured after 12 to 24 h, 0.89 (0.77–1.0) for S100b measured after 24 to 48 h, and 0.84 (0.65–1.0) for S100b measured after 48 to 72 h. The cut-offs with corresponding predictive values are presented in Table 3.

### Blood Glucose and Lactate Levels

The AUC for prediction of 30-day mortality was 0.82 (0.63–1.0) for blood glucose measured at admission. The AUC for prediction of 30-day mortality was 0.69 (0.50–0.87) for lactate measured at admission and 0.74 (0.54–0.93) for lactate measured 12 h after admission.

## Discussion

The primary finding of this study was that NSE and S100b levels measured 12 to 72 h after admission in children resuscitated from CA were strong predictors of 30-day mortality and of poor neurologic outcome after 180 days.

NSE is mainly found in neuronal and neuroendocrine cells and S100b in astrocytes and Schwann cells [18, 19]. Following cerebral injury these cells are damaged and the biomarkers are released to the blood. Their level in serum is related to the extent of cerebral injury and therefore potentially able to predict outcome [1, 15]. NSE is suggested to be used for as part of a multimodal prognostication strategy after CA, where the majority of mortality can be attributed to anoxic–ischemic neurological injury [7]. S100b is mainly applied after traumatic brain injury related to diffuse axonal injury [10]. However, extra-central nervous system sources can confound biomarker analysis [3]. Increased serum levels of NSE can be caused by hemolysis as well as presence of small cell lung carcinoma and neuroendocrine tumors [6]. Increased serum levels of S100b is reported after cardiac surgery as well as after hypoperfusion in patients with sepsis [3].

Three previous studies have investigated the predictive ability of NSE and S100b in children with CA. The 30-day survival rate was 62% in the present study, which is comparable to the survival rates reported in the three other studies. The largest and most recent study by Kramer et al. included 95 children with CA from two departments of congenital heart disease and congenital heart surgery [1]. The primary outcome was PCPC score at discharge, and the study reported AUC’s from 0.73 to 0.83 for NSE and from 0.78 to 0.82 for S100b measured at 24 to 72 h. The lower predictive ability of the biomarkers compared to the present study may be explained by one or more of the following factors. In contrast to the present study (which included one patient with congenital heart disease), Kramer et al. primarily included post-surgical patients with congenital heart disease. In this population, death caused by circulatory failure is likely to be more prevalent compared to an all-comer population of mixed causes of CA, where death caused by neurologic injury may be more prevalent. Further, in the study by Kramer et al., a large proportion of patients underwent cardio-pulmonary bypass, and elevated NSE levels caused by hemolysis may have confounded the results. Finally, in a larger sample size, the higher likelihood of outliers will generally lead to lower predictive values for a given biomarker. A prospective study by Topjian et al. included 35 patients resuscitated after CA of mixed origin [3]. The study reported an area under the ROC curve of 0.85 for NSE measured after 48 h versus poor neurological outcome defined as the PCPC score before discharge. In 43 children with resuscitated CA, Fink et al. reported an AUC of 0.79 for NSE versus mortality and an AUC of 0.91 for S100b versus mortality [15]. In contrast to the present study, biomarker values from multiple timepoints were pooled for assessment of ROC.

The paper by Fink et al. reported an NSE cut-off value of 53 μg/L at a false-positive rate of 0 when measured after 24 h and a cut-off value of 77 μg/L when measured after 48 h in children after CA [15]. In contrast, Kramer et al. reported higher cut-off values of NSE at 133 μg/L when measured after 24 h, 118 μg/L when measured after 48 h, and 112 μg/L when measured after 72 h [1]. The higher cut-off values, as well as the decreasing trend of the cut-off values, may be explained by hemolysis occurring during index heart surgery, i.e., prior to CA.

Contemporary guidelines for post-resuscitation care in adults suggest the use of NSE as part of a multimodal prognostication strategy. Here, an NSE cut-off above 60 μg/L after 48 and/or 72 h is suggested as a marker of poor prognosis [7]. Accordingly, the suggested cut-off values by the present article correspond well with the suggested cut-off value for adults. Importantly, adult guidelines emphasize that a multimodal prognostication approach should be applied to limit the risk of a false-positive, i.e., withdrawal in a patient with a potential good outcome. The need to minimize the risk of false-positive prognostication is no less important in pediatric CA, and it is unlikely that any one test will be sufficiently accurate for prognostication. However, NSE may prove an important part in future multimodal prognostication strategies. Current guidelines do not recommend the use of S100b for prognostication after adult CA, as the predictive ability is inferior compared to NSE [7]. This corresponds to the results of the present paper. Guidelines for prognostication after resuscitated CA in children simply suggest that biomarkers may be indicative, but that cut-off values remain unknown [20].

Compared to other modes of prognostication after CA biomarkers have the advantage of being independent of sedatives which often confound outcome prediction in resuscitated patients treated in the intensive care unit [7, 21]. Further, biomarkers have the advantage that the clinical team can be blinded for the results, and as such, biomarkers may serve as potential outcome markers in future clinical trials.

The presented results should be interpreted after consideration of the following limitations. The study was observational in design, and a sample size of 32 children limits the probability of identifying outliers. The presented predictive abilities of the biomarkers are likely to be lower in a larger population with a higher likelihood of outliers, and the cut-off value will need to be evaluated in larger prospective studies prior to clinical application. There are many causes of CA and it is likely that the predictive ability of the biomarkers may depend on various other factors connected to the specific cause of CA. The present sample size is too small for conducting analyses on subgroups. Accordingly, the results should be considered hypothesis-generating.

## Conclusion

In children resuscitated from CA, NSE, and S100b analyzed 12 to 72 h after admission are highly associated with 30-day mortality, and the biomarkers appear to be strong predictors of mortality. Future large-scale prospective studies are needed to further define cut-off points and to potentially combine biomarkers with clinical risk scores.
